# Sendai Virus, a Strong Inducer of Anti-Lentiviral State in Ovine Cells

**DOI:** 10.3390/vaccines8020206

**Published:** 2020-04-29

**Authors:** Lorena de Pablo-Maiso, Irache Echeverría, Sergio Rius-Rocabert, Lluís Luján, Dominique Garcin, Damián de Andrés, Estanislao Nistal-Villán, Ramsés Reina

**Affiliations:** 1 Department of Animal Health, Institute of Agrobiotechnology (CSIC-Government of Navarra), 31192 Mutilva, Navarra, Spain; lorena.depablo@unavarra.es (L.d.P.-M.); irache.echeverria@unavarra.es (I.E.); damian.deandres@csic.es (D.d.A.); 2 Microbiology Section, Departamento Ciencias Farmacéuticas y de la Salud, Facultad de Farmacia, Universidad CEU San Pablo, CEU Universities, Boadilla del Monte, 28668 Madrid, Spain; ser.rius.ce@ceindo.ceu.es (S.R.-R.); estanislao.nistalvillan@ceu.es (E.N.-V.); 3 CEMBIO (Centre for Metabolomics and Bioanalysis), Facultad de Farmacia, Universidad CEU San Pablo, CEU Universities, Boadilla del Monte, 28668 Madrid, Spain; 4 Department of Animal Pathology, University of Zaragoza, 50013 Zaragoza, Spain; lluis.lujan@unizar.es; 5 Department of Microbiology and Molecular Medicine, University of Geneva, 1211 Geneva, Switzerland; dominique.garcin@unige.ch; 6 Instituto de Medicina Molecular Aplicada (IMMA), Universidad CEU San Pablo, Pablo-CEU, CEU Universities, Boadilla del Monte, 28003 Madrid, Spain

**Keywords:** small ruminant lentivirus, Sendai virus, innate immunity, interferon, APOBEC3

## Abstract

Small ruminant lentiviruses (SRLVs) are widely spread in the ovine and caprine populations, causing an incurable disease affecting animal health and production. Vaccine development is hindered owing to the high genetic heterogeneity of lentiviruses and the selection of T-cell and antibody escape mutants, requiring antigen delivery optimization. Sendai virus (SeV) is a respiratory paramyxovirus in mice that has been recognized as a potent inducer of innate immune responses in several species, including mouse and human. The aim of this study was to stimulate an innate antiviral response in ovine cells and evaluate the potential inhibitory effect upon small ruminant lentivirus (SRLV) infections. Ovine alveolar macrophages (AMs), blood-derived macrophages (BDMs), and skin fibroblasts (OSFs) were stimulated through infection with SeV encoding green fluorescent protein (GFP). SeV efficiently infected ovine cells, inducing an antiviral state in AM from SRLV naturally-infected animals, as well as in in vitro SRLV-infected BDM and OSF from non-infected animals. Supernatants from SeV-infected AM induced an antiviral state when transferred to fresh cells challenged with SRLV. Similar to SRLV, infectivity of an HIV-1-GFP lentiviral vector was also restricted in ovine cells infected with SeV. In myeloid cells, an M1-like proinflammatory polarization was observed together with an APOBEC3Z1 induction, among other lentiviral restriction factors. Our observations may boost new approximations in ameliorating the SRLV burden by stimulation of the innate immune response using SeV-based vaccine vectors.

## 1. Introduction

Small ruminant lentiviruses (SRLVs) are widely spread in sheep and goats throughout the world, causing a multiorgan disease affecting animal welfare and production. SRLV comprises Visna Maedi virus (VMV), the first lentivirus discovered and a good model for HIV studies (as recently described for the integrase supramolecular assembly [1]), and the caprine arthritis encephalitis virus (CAEV), which can be used to generate lentiviral vectors for gene transfer [2].

Vaccine-mediated immunization against SRLV is ineffective in the same way as it remains elusive for other lentiviruses such as HIV [3]. Control strategies to protect animals beyond specific animal management of seropositive individuals are not available [4]. Current control programs present some difficulties such as the ability to perform efficient and reliable serological tests to detect the complete antigenic spectrum that SRLV exhibits in nature, or the difficulty in detecting low antibody responders and delayed seroconversion [5,6].

SRLV can target and stably infect macrophages, controlling cellular response and modulating differentiation pathways and cytokine secretion in order to maintain a sustained replication [7,8]. In contrast, pro-inflammatory macrophages (classically activated or M1) are known as a differentiation state that can restrict lentiviral replication in humans [9] and also in sheep [8]. However, the underlying mechanisms of how they function are not fully characterized.

Induction of humoral and cellular immune responses upon challenge with homologous SRLV vaccine strains can confer partial protection in animals. This protective effect can be quantified as a reduced viremia and delayed disease development [10]. However, the efficacy of these vaccines upon challenge with heterologous genotypes, which may be present in field infected animals, is expected to be limited. Furthermore, long-term protection is highly queried as escape mutants are expected [11].

Stimulating the innate immune response may relieve these limitations by inducing interferon (IFN) production, thereby triggering antiviral responses in the absence of specific recognition of viral epitopes. This stimulation may activate the cell defensive barriers, preventing infection by incoming viruses as well as controlling chronically infected cells by reducing the viral load. In addition, this stimulation can induce better antigen processing and presentation. Several IFN-induced proteins are considered responsible for the species-specific restriction of lentiviruses, including TRIM5α, APOBEC3, and Tetherin, which are able to block the virus at different steps during the viral replication cycle [12]. Indeed, recent research based on next generation sequencing has identified a series of interferon-stimulated genes (ISGs) related to SRLV infection or disease development, such as RIG-I or SAMHD1 [13].

Sendai virus (SeV) is a paramyxovirus that was initially described as a respiratory mouse adapted virus. SeV is currently recognized as a potent inducer of the interferon antiviral response in various animal models and also as an efficient vector for airway gene transfer [14]. Pathogen associated molecular patterns (PAMPs) present during SeV infection, such as double stranded RNAs, are sensed by cellular pattern recognition receptors (PRRs) (mainly RIG-I like receptors) inducing intracellular signaling, which triggers the transcription of antiviral and immune-stimulated genes [15]. This immune activation has prompted the development of SeV-derived vectors for vaccination [16], inducing a well characterized type-I IFN antiviral response. Production of type I IFNs drives further gene induction in a secondary signaling cascade, which amplifies and regulates the cellular antiviral state. Type-I IFN-primed cells can act as a barrier against virus replication, particularly in lentivirus infected cells, in which type-I IFN response is inhibited [17]. In fact, SeV-derived vaccines are currently being tested against a series of pathogens including lentiviruses such as HIV-1 [18].

Here, we hypothesize that stimulating cellular PRRs and antiviral responses using SeV can control SRLV infection in ovine cells. Furthermore, such stimulation could restore cell defenses and recover the intrinsic immune response against SRLV, aiming for an eventual viral clearance. SRLV susceptible cells, such as fibroblasts and blood-derived, as well as alveolar macrophages, can be efficiently infected by SeV. The innate response induced after SeV infection was evaluated by mRNA relative quantification of M1/M2 ovine macrophage differentiation markers as well as lentivirus restriction factors. The results revealed a proinflammatory pattern in ovine myeloid cells and reduced SRLV DNA and RNA levels and virus production in both naturally and in vitro infected cells. This antiviral state likely involves type-I IFN induction.

These findings broaden our understanding of the interplay between the ovine innate immune response and SRLV infection, opening new insights into the development of new prophylactic and therapeutic strategies.

## 2. Materials and Methods 

### 2.1. Cells and Viruses

Alveolar macrophages (AMs) of nine SRLV naturally-infected sheep were obtained by bronchoalveolar lavage centrifugation at 800× *g* for 10 min. Cell pellets were seeded in 12-well plates and incubated in Roswell Park Memorial Institute (RPMI) complete medium (1% of vitamins, 10 mM sodium pyruvate, 1% non-essential amino acids, 1% l-glutamine, 50 µm β-mercaptoethanol, 1% antibiotics/antimycotics mix; (Sigma Aldrich, St. Louis, MO, USA)) supplemented with 10% heat-inactivated fetal bovine serum (FBS; Sigma Aldrich, St. Louis, MO, USA), as previously described [19].

Peripheral blood mononuclear cells (PBMCs) from SRLV-free sheep, confirmed by serology (Eradikit™ SRLV, In3Diagnostic, Torino, Italy) and PCR [20,21], were seeded in 12-well plates and adherent cells were allowed to differentiate into blood-derived macrophages (BDMs) for twelve days of culture in RPMI complete medium supplemented with 10% heat-inactivated FBS [22].

Primary cultures of ovine skin fibroblasts (OSF) were obtained from SRLV-seronegative animals as previously described [23] and used for in vitro infection. T-immortalized goat embryo fibroblasts (TIGEF; kindly provided by Dr. Y. Chebloune, University of Lyon, France) and goat synovial membrane cells (GSM-T; kindly provided by Dr. S. Valas, Anses Niort Laboratory, Niort Cedex, France) were grown in Dulbecco’s modified Eagle’s medium (DMEM) supplemented with 10% heat-inactivated FBS, 1% l-glutamine, and 1% antibiotics/antimycotics mix (Sigma Aldrich, St. Louis, MO, USA).

SRLV viral stocks from the genotype A (EV1 strain) [24] and from the genotype B (496 strain) [25] were titrated on OSF in 96-well culture plates using the Reed–Müench method and used in in vitro infections, as specified [26].

SeV-GFP vector encoding the green fluorescent protein (GFP) was grown in 10 day embryonated eggs for 72 h and stocks of 10^9^ plaque-forming units (PFU)/mL obtained, as previously described [27].

Recombinant Vesicular Stomatitis virus expressing GFP (VSV-GFP), used as a reporter of infection, was grown in Vero cells for 48 h and clarified for 15 min by centrifugation at 10,000× *g*. The virus was titrated in Vero cells following the Reed–Muënch method [26].

VSV-G pseudotyped HIV-GFP vector (kindly provided by Dr. Towers, University of London, United Kingdom) was produced in 293-T cells by transfection with three plasmids using JetPrime (PolyPlus, Illkirch-Graffenstaden, France), as described [28]. Supernatants obtained 48 h after transfection were used at different dilutions as specified in transduction experiments.

HIV-1 GFP-based vector infectivity was analyzed by quantifying GFP integrated into cellular DNA, because SeV-GFP was not integrated into the chromosome of the host. GFP copies were quantified by qPCR in an AriaMx Realtime PCR System (Agilent Technologies, Santa Clara, CA, USA), following standard procedures [29].

### 2.2. Cell Infection and Virus Quantification

AM, BDMs, and OSF were infected with SeV-GFP virus vector at different multiplicity of infection (MOI) and infectivity was determined by flow cytometry (FACScalibur, BD Bioscience, San Jose CA, USA) and using fluorescence microscopy 48 h after infection (Nikon Eclipse TE300) to detect virus-encoded GFP fluorescence. Prior to assessment by flow cytometry, cells were treated with trypsin to ensure a single-cell suspension optimal for analysis and fixed with 0.5% paraformaldehyde (Sigma Aldrich, St. Louis, MO, USA). 

SeV-infected BDM and OSF were further infected with SRLV at an MOI of 0.5, as previously described [30]. After 16 h, medium was replaced, cells washed with phosphate-buffered saline (PBS) (Sigma Aldrich, St. Louis, MO, USA), and further incubated with DMEM 2% FBS. DNA was obtained from infected cells after 16 h according to the manufacturer’s instructions (E.Z.N.A. tissue/blood kit OMEGA Bio-tek, Norcross, GA, USA) and SRLV copies were determined using real time PCR with different TaqMan probes for Ov496 and EV1 strains, as described [25]. RNA was obtained from cells 48 h after SRLV infection by chloroform-isopropanol precipitation, as previously described [31]. RNA was treated with TurboDNaseI (Ambion, ThermoFisher Scientific, Waltham, MA, USA) and purified by extraction with phenol acid, chloroform, and ethanol precipitation. Then, 1 µg of total RNA was retrotranscribed using PrimeScript RT Kit (Takara, Kioto, Japan) and oligo-dT primers. Viral cDNA from P25 capsid protein was quantified by real time (rt)-PCR using previously described primers [21].

Virus production was evaluated according to retrotranscriptase (RT) activity in supernatants by SYBR Green based real time PCR enhanced reverse transcriptase assay (SG-PERT) [32]. Briefly, virus particles from 5 µL of supernatant were lysed (0.25% Triton X-100, 50 mM KCl, 100 mM Tris-HCl pH 7.4 and 40% glycerol) and viral RT was incubated with a master mix containing RNA from bacteriophage MS2 (Sigma-Aldrich, St. Louis, MO, USA) and RNAases inhibitors (RiboLock, ThermoFisher Scientific, Waltham, MA, USA) for 20 min at 42 °C. After retrotranscription, the resulting MS2 cDNA was subjected to real time quantification using described primers and protocols [32]. A standard curve, consisting of dilutions of titrated SRLV stocks, was constructed and performed with samples for each analysis for quantification.

As AM were obtained from SRLV-naturally infected animals, SRLV viral DNA was quantified 48 h after SeV-GFP infection and RNA as well as RT activity through SG-PERT were quantified at 72 h after infection with SeV-GFP. Supernatants obtained 48 h after SeV-GFP infection were also transferred to fresh OSF and cultured for a further 24 h. Then, OSFs were infected with SRLV at 0.5 MOI for 16 h, and cells were washed twice with PBS and incubated with DMEM 2% FBS. SRLV production was evaluated by SG-PERT, as described above, in supernatants 72 h after infection.

### 2.3. mRNA Relative Quantification

Amplification of different ovine restriction factors (A3Z1, A3Z2Z3, OBST2, TRIM5α, and SAMHD1) and of markers of the ovine macrophage differentiation M1 and M2 pathways (A3Z1, TNF-α, MR, and DC-SIGN) was performed by quantitative PCR on an AriaMx Realtime PCR System (Agilent Technologies, Santa Clara, CA, USA), using SYBR Premix Ex Taq (Takara, Kyoto, Japan) with primers previously described [28,30,33].

Primer3 software [34] was applied to design specific primers for SAMHD1 transcript variant X1 (Fw5′-GAGAACGAAGCTGCTTAATTGTATCC-3′; Rv5′ GAGGTGTGTCGATGATTCGGA-3′) and OBST2 (Fw 5′-CGTGGACGGCCTCCAAG-3′, Rv 5′-TGGCAGCTTCGGCTTCC-3′). Four different housekeeping genes (GAPDH, G6PDH, YWHAZ, and β-actin) were evaluated. Data analyzed with NormFinder and GeNorm software showed β-actin as the most stable gene for relative quantification (2^−ΔCt^ or 2^−ΔΔCt^ methods). RIG-I expression was quantified with designed primers based on the predicted Ovis aries DDX58 sequence from Genbank XM_004005323.3 (Fw 5′-GCTGACGGCCTCAGTTGGT-3′, Rv 5′-TCGAGAGAAGCACACAGTCTGC-3′).

### 2.4. Type-I IFN Bioassay

In order to quantify the IFN bioactivity present in the supernatant of infected cells, we adapted the traditional IFN bioassay, to be used for ovine cells. Briefly, the supernatant from SeV-infected ovine AM was serially diluted in DMEM medium supplemented with 10% FBS and 1% streptomycin and penicillin (Sigma Aldrich, St. Louis, MO, USA). This supernatant was added to OSF cells that were seeded at 2 × 10^4^ per well in 96-well plates the day before. These OSF cells were incubated at 37 °C for 24 h. After incubation, supernatants were removed and OSF cells were infected with recombinant VSV-GFP at a MOI of 0.01 and incubated at 37 °C. Then, 18 h after infection, VSV-GFP infected wells were detected by the expression of green fluorescence and quantified by the use of a Varioskan Flash plate reader (ThermoFisher Scientific, Waltham, MA, USA) with an excitation wavelength of 480 nm and emission of 518 nm. The assay was performed with triplicate dilutions and 12 measurements per well [35].

### 2.5. Statistical Analysis 

Statistical analysis was carried out using PRISM version 5.01 (GraphPad Prism, GraphPad Software Inc., San Diego, CA, USA) and SPSS Software v.23 (IBM Company, New York, NY, USA). Statistical significance was assigned to *p* < 0.001 (***), *p* < 0.01 (**), or *p* < 0.05 (*). After testing normal distribution of the data, T-Student or Mann–Whitney tests were applied when appropriate, as indicated.

## 3. Results

### 3.1. SeV Infection Is Highly Efficient in Ovine Cells

**Figure 1:**
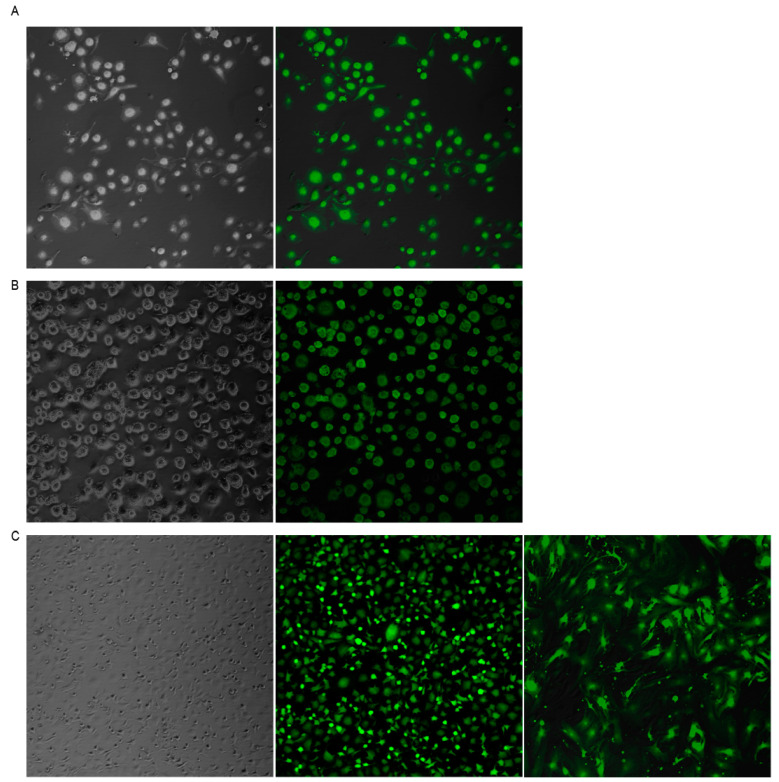
Sendai virus (SeV)-green fluorescent protein (GFP) infection of ovine cells. Fluorescence microscopy images of alveolar macrophages (AMs) (**A**), blood derived macrophages (BDMs) (**B**), and ovine skin fibroblasts (OSFs) (**C**) infected with Sendai virus vector expressing the GFP (right panel) at a multiplicity of infection (MOI) of 10. Bright field images are shown in the left panel. The three cell types and all cells in the three cultures are GFP-positive. Ovine fibroblasts remained GFP-positive after 13 in vitro culture passages ((**C**), third image).

In order to test whether SeV can enter and replicate in ovine cells, different MOI were tested in OSF (Supplementary Figure S1). Alveolar macrophages (AMs) (Figure 1A) and blood-derived macrophages (BDMs) (Figure 1B), as well as skin fibroblasts (OSFs) (Figure 1C) primary cell cultures, were infected with SeV-GFP. Infection was very efficient 48 h after infection in the three cell types tested, reaching 100% of GFP positive cells.

### 3.2. SeV Infection Induced Stable GFP Expression in Ovine Cells

GFP expression was stable in OSF for at least 13 in vitro cell passages (Figure 1C). However, transfer of supernatants from SeV-infected cells to fresh cultures resulted in GFP-negative events, indicating that the virus was not produced in ovine cells (Supplementary Figure S2). Furthermore, PCR amplification using GFP-specific primers from genomic DNA was negative in all cells tested, indicating a lack of SeV-GFP integration into the host genome.

### 3.3. SeV Infection Induces Proinflammatory Responses in Ovine Cells

**Figure 2:**
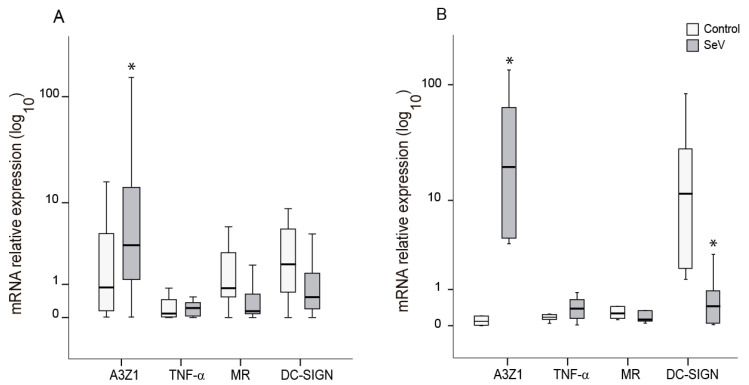
Differentiation markers in ovine myeloid cells infected with Sendai virus (SeV). Relative expression of M1 (A3Z1, TNF-α) and M2 (MR, DC-SIGN) differentiation markers measured in alveolar macrophages (**A**) and blood derived macrophages (**B**). Values are the median (±interquartile range) of at least three independent experiments. * *p* < 0.05 (paired Mann–Whitney U Test).

Markers of the proinflammatory (M1) and anti-inflammatory (M2) differentiation pathways were evaluated in ovine myeloid cells (AM and BDM) upon infection with SeV. In both cases, SeV infection induced an M1-like pattern characterized by high A3Z1 and low MR expression (Figure 2). A3Z1 was induced in AM and BDM (Figure 2A,B) and DC-SIGN was additionally decreased in BDM (Figure 2B). The high variability in the induction levels between animals could be attributed to genetic and immune status differences in each of the animals.

### 3.4. SeV-Infected Cells Reduced Permissibility to SRLV Infection

**Figure 3:**
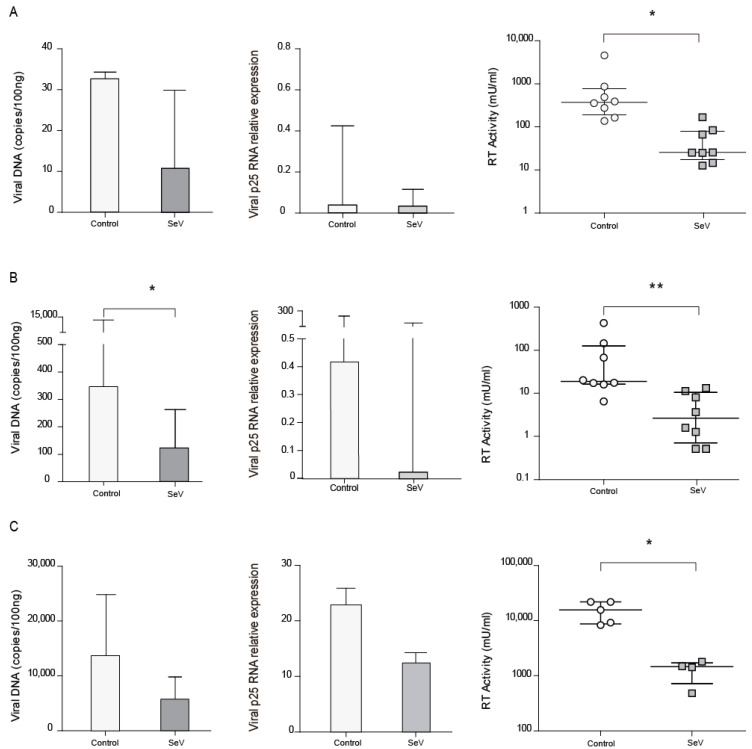
Small ruminant lentivirus (SRLV) replication in ovine cells in the context of SeV. SRLV restriction in ovine alveolar macrophages from chronically infected animals (**A**), or non-SRLV infected animals’ blood derived macrophages (**B**) and skin fibroblasts (**C**) that were mock, or Sendai virus (SeV) infected and challenged later on with SRLV. SRLV viral DNA (left panel), Gag-p25 mRNA relative expression (mid panel), and retrotranscriptase (RT) activity (right panel) was measured in AMs of infected animals or BDMs or OSFs from uninfected animals infected with SeV at an MOI of 10 (grey bars). BDMs and OSFs were further infected with SRLV at an MOI of 0.5 (white bars). Viral DNA was measured at 16 h post-infection, p25 mRNA was measured at 48 h post-infection, and RT activity was measured by SYBR Green based real time PCR enhanced reverse transcriptase assay (SG-PERT) in clarified supernatants at 72 h post-infection. Data shown are the median (±interquartile range) and differences were analyzed using the Wilcoxon paired test (* *p* < 0.05, ** *p* < 0.01).

As the M1 differentiation pathway has been reported to inhibit SRLV infection, AMs from naturally SRLV-infected animals were infected with SeV and checked for SRLV viral DNA and RNA as well as RT activity in the supernatant. SRLV viral DNA and p25 gene expression were non-significantly reduced (*p* = 0.24 and *p* = 0.31, respectively), however, viral production was significantly (*p* < 0.05) inhibited in AM; Figure 3A.

To extend these observations, BDMs from uninfected animals were experimentally infected with SeV-GFP, in order to achieve innate antiviral response, and subsequently infected with SRLV in vitro. BDMs showed lower virus DNA levels (*p* < 0.05) and a slight reduction in viral RNA together with a reduced viral production when infected with SeV (Figure 3B).

In addition to immune cells, permissive skin ovine fibroblasts, routinely used to propagate SRLV in vitro, were also stimulated with SeV-GFP and infected with SRLV. SRLV viral DNA only exhibits a trend to be lower (*p* = 0.06) and RNA levels were not significantly altered. However, SRLV viral production in the supernatant was significantly inhibited (Figure 3C and Supplementary Figure S3).

These results were extended to other SRLV permissive cell lines, such as TIGEF and GSM-T cells, showing high rates of infection with SeV-GFP and significant restriction of SRLV replication and viral DNA levels (Supplementary Figure S4).

### 3.5. Ovine Cells Infected with SeV-GFP Inhibit HIV-1-GFP Vector Infectivity

**Figure 4:**
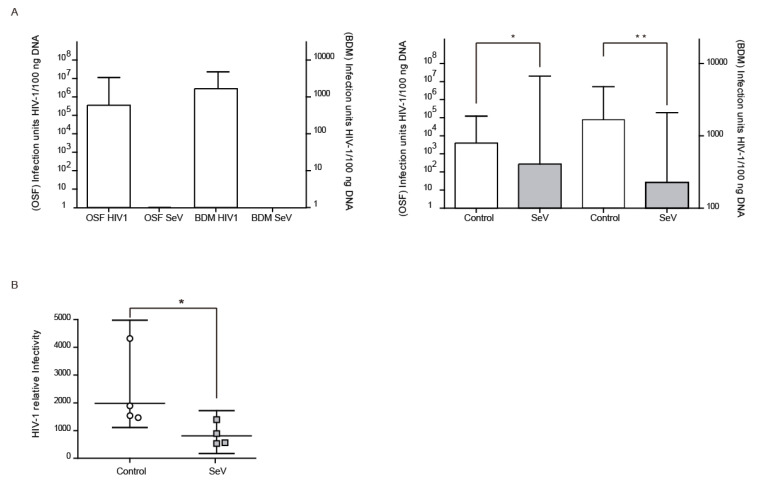
Pseudotyped Human Immunodeficiency virus (HIV-1) restriction after Sendai virus (SeV) infection. ((**A**), left panel) HIV-1-GFP proviral load in ovine skin fibroblasts (OSFs; left axis) and blood-derived macrophages (BDMs; right axis) infected with HIV-1 GFP-based vector or SeV-infected. Values represent the geometric mean copy values (±95% confidence interval (CI)) per 100 ng of total DNA. ((**A**), right panel) GFP proviral copies measured in uninfected and SeV-infected OSF and BDM transduced with HIV-1 GFP vector. Values are the geometric mean copies (±95% CI) per 100 ng of total DNA. Differences were statistically analyzed using unpaired T test (one-tailed), * *p* < 0.05. (**B**) Relative infectivity in 293-T cells of HIV-1-GFP pseudovirus produced in uninfected 293-T cells (control; white bars) or infected with SeV (SeV; grey bars) in fresh 293-T cells. Values are the geometric mean (±95% CI) of at least three independent experiments. Differences were statistically analyzed using paired T test (one-tailed), * *p* < 0.05, ** *p* < 0.01.

Beyond SRLV, the antiviral state induced in ovine cells after SeV infection may also affect the infectivity of heterologous lentiviruses such as HIV. VSV-G pseudotyped HIV-1-GFP vector infectivity could be analyzed in OSF and BDM by qPCR of the recombinant HIV-derived GFP integrated gene (Figure 4A, left panel). OSF and BDMs previously infected with SeV-GFP also showed reduced HIV-1 vector infectivity (Figure 4A, right panel). Furthermore, HIV-1 vector production was less efficient in 293-T cells previously infected with SeV-GFP in single cycle infection experiments (Figure 4B).

### 3.6. Restriction Factors Induced after SeV Infection in Ovine Cells

**Figure 5:**
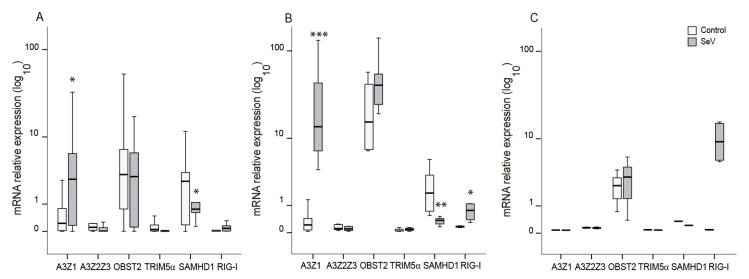
Lentiviral restriction factors mRNA expression in ovine cells after infection with Sendai virus (SeV). Ovine APOBEC3 proteins (A3Z1 and A3Z2Z3), tetherin (OBST2), TRIM5α, SAMHD1, and RIG-I mRNA expression was quantified in control (white) and SeV-infected (grey) ovine alveolar macrophages (**A**), blood derived macrophages (**B**), and skin fibroblasts (**C**). Values are the median (±interquartile range) of at least three independent experiments, * *p* < 0.05, ** *p* < 0.01, *** *p* < 0.001 (paired Mann-Whitney U test).

Ovine myeloid cells (AM and BDM) infected with SeV increased A3Z1 mRNA expression among described restriction factors against lentivirus infection. Other APOBEC3 proteins or other restriction factors such as tetherin, SAMHD1, or TRIM5α were not induced upon SeV infection (Figure 5A,B). Indeed, SAMHD1 expression was lower in SeV in myeloid-infected cells.

Additionally, the expression of an interferon stimulated gene, retinoic acid inducible gene-I (RIG-I), increased in BDMs infected with SeV as compared with uninfected cells. This induction was also observed as a trend in OSF (*p* = 0.11; Figure 5C).

### 3.7. SeV Infection May Induce Local Resistance to SRLV

As myeloid cells may induce antiviral responses through autocrine and paracrine mechanisms, SRLV production was evaluated in OSFs cultured with supernatants from AMs previously infected with SeV-GFP. Viral infection is recognized by infected cells, triggering the IFN-β pathway, which leads to the transcriptional expresion of the IFNB1 gene as well as other genes. Upon translation, IFN-β (as well as other type I IFN) is secreted and can bind to the type I IFN receptor (IFNAR) and trigger a second pathway, which leads to the expression of many genes. An estimation of the paracrine effect triggered by secreted type I IFN can be calculated by different means.

**Figure 6:**
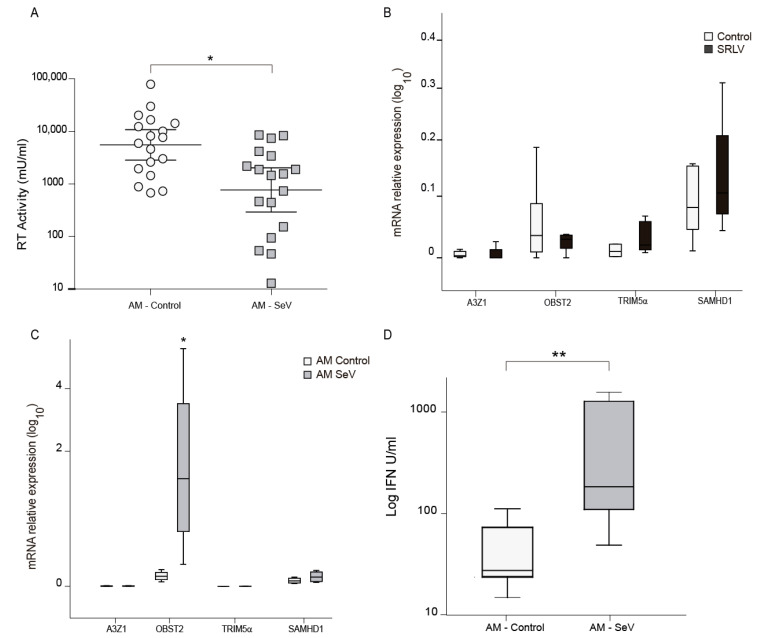
Antiviral activity induction after infection with Sendai virus (SeV). (**A**) RT activity in ovine skin fibroblasts (OSFs) cultured with supernatants from alveolar macrophages (AMs), infected or not with SeV, were infected with SRLV after 24 h of supernatant treatment. SRLV virus production was measured as retrotranscriptase (RT) activity in the supernatant at 72 h post infection. Data shown are the geometric mean ±95% CI of at least three independent experiments. Differences were statistically analyzed using unpaired T test, * *p* < 0.05. (**B**) Relative mRNA expression of the restriction factors after infection with SRLV. Data shown are the mean ± SEM of at least three independent experiments. * *p* < 0.05 (paired Mann–Whitney U test). (**C**) Relative mRNA expression of restriction factors: ovine APOBEC3Z1 (A3Z1) tetherin (oBST2), TRIM5α, and SAMHD1 measured by quantitative RT-PCR in ovine skin fibroblast (OSF) cultured with supernatants from AM control or AM infected with SeV. Values are the median (±interquartile range) of at least three independent experiments. * *p* < 0.05 (Mann–Whitney paired U Test). (**D**) Type-I interferon (IFN) quantification measured by an ovine-adapted IFN bioassay using supernatants from AM control or infected with SeV. Data shown are the median ±interquartile range of at least three independent experiments. * *p* < 0.05, ** *p* < 0.01 (Mann–Whitney paired U test).

Quantification of the antiviral effect in supernatants can be stimated by measuring the antiviral effect on SRLV RT activity. Supernatants from SeV primed ovine AM were transferred into OSF cells to trigger an antiviral state in them. A control to detect the absence of SeV present in the supernatant was performed (data not shown). Then, 18 h later, OSF cells were challenged with SRLV and viral RT activity was measured. In this way, decreased RT activity is a sign of reduced viral production, indicating that the resistance acquired upon SeV infection can be transferred to proximal cells (Figure 6A). As AMs were naturally infected with SRLV and this may influence mRNA gene expression, RNA from SRLV experimentally infected OSF was also evaluated. Invariable A3Z1 and BST2 mRNA expression levels were found, suggesting cell-specific induction of BST2 in SeV-infected OSFs (Figure 6B).

Consequently, mRNA expression of some interferon-stimulated genes that can act as restriction factors against SRLV was analyzed. Increased ovine BST2 (oBST2) expression was found after supernatant treatment (Figure 6C). Aiming at revealing the mechanisms, we developed an ovine specific IFN biassay that quantifies the biological activity of IFN. Supernatants from SeV-infected ovine AM in culture were tested in a type-I IFN bioassay for the induction of an antiviral state in fresh OSF cells. OSF cells treated with this supernatants will trigger an antiviral program if the supernatant contains IFN. Challenging later on the OSF with a virus-like VSV-GFP will allow to determine the protection against VSV-GFP by the quantification of green fluorescence protein. Supernatants from SeV-infected AM showed a clear interference, indicative of the presence of type I IFN (Figure 6D).

## 4. Discussion

New control strategies are needed not only in veterinary, but also in human medicine against lentivirus infections. Vaccination strategies explored so far against SRLV, one of the most prevalent infections in livestock, have been proven to be inefficient. Currently, available SRLV control strategies rely on limited serological diagnosis owing to the antigenic heterogeneity of strains. Indeed, genetic as well as antigenic variations are wider than previously thought, with recent descriptions of novel genotypes and subtypes enlarging SRLV’s antigenic spectrum, a concern that jeopardizes the development of highly sensitive diagnostic tests and effective vaccines [36], raising the question of whether heterologous strains could be restricted by the adaptive immune responses. Accordingly, humoral and cellular immune responses are generally genotype-specific against natural and experimental SRLV infections, which invalidates vaccine cross-protection [37,38,39]. Immunization experiments have induced specific humoral and cellular immune responses that conferred only partial protection against challenge with homologous strains [40]. Finally, antiretroviral therapy is not an affordable option in sheep owing to economic (the high price of the drugs) restrictions.

This study introduces the induction of innate antiviral responses using a recombinant Sendai virus expressing GFP in SRLV permissive cells, such as macrophages (tissue resident and circulating) and skin fibroblasts. Infection resulted in virtually 100% of GFP-positive cells (Figure 1), which is by far higher than the rates reached with plasmid transfection or lentiviral transduction in ovine primary cultures [41]. SeV uses sialic acid-containing molecules as receptors that are present in the surface of most cell types [42], including the ovine cells tested in this work. This high efficiency is particularly interesting in the case of macrophages, as they are considered cells hard-to-transfect or transduce [41]. SeV-driven GFP expression was stable in OSF for at least 13 tissue culture passages, reflecting a stable SeV genome replication and recombinant protein expression. In addition, SeV was not integrated or produced by the ovine cells, as the supernatant transferred from SeV-infected cells to fresh cells resulted in no GFP expression, in agreement with a previous in vivo report [19].

SRLV inhibition was evidenced at different steps of the virus replication cycle depending on the cell type analyzed. Ovine myeloid cells (AM and BDM) infected with SeV exhibited an M1-like differentiation profile upon infection that can explain the reduced SRLV replication observed (Figure 2). M1 differentiation was more evident in BDM than in AM, as the latter were already M2-like differentiated cells [8,43], and re-differentiation to M1 may require longer stimulation periods and more than one stimulation cycle [8,9].

A3Z1 is a host cytidine deaminase that mutates DNA viral genomes before integration, thereby restricting infection. A3Z1 transcript expression was elevated in AM and BDM after SeV infection. This is in accordance with the restrictive pattern observed against SRLV, because SRLV viral DNA production and virus generation decrease in SeV-infected BDMs. However, SeV infection of SRLV naturally infected AM restricted SRLV production and showed no differences at the viral DNA level. This discrepancy could be explained by the low efficiency of primers in detecting strains present in the field [44]. Primers used may have missed the natural circulating strain infecting the flock of origin, while efficiently amplifying the SRLV strain used in BDM in vitro infections.

Similar to the human orthologue A3A, the results presented in this manuscript suggest that ovine A3Z1 protein seems to play a major role in myeloid cells (M1-macrophages and monocytes [30]), and not in other lentivirus permissive cells, such as fibroblasts, where other restriction factors may exert greater antiviral activity [45]. Surprisingly, mRNA expression of SAMHD1 (another lentiviral restriction factor) was downregulated in ovine myeloid cells infected with SeV. SAMHD1 acts at pre-integration steps of the lentiviral replication cycle through dNTPs and/or viral RNA degradation [46]. In addition, SAMHD1 expression could affect innate immune responses [47]. Infection of ovine cells by SeV could counteract this activity by reducing SAMHD1 expression.

On the other hand, ovine fibroblasts respond to SeV infection by restricting SRLV production in vitro without a significant reduction in the viral DNA levels (Figure 3). The different antiviral programming that SeV-GFP induced in OSF is characterized by faint inductions of RIG-I and BST2 and not the expression of A3Z1, and may account for this restriction pattern (Figure 5). While RIG-I is a typical ISG involved in viral dsRNA recognition and the induction of IFN, and antiviral responses, BST-2, as a transmembrane protein, is able to block the budding of emerging virus particles from the plasma membrane, thereby reducing cell-to-cell transmission without affecting other restriction sites or signaling events [48]. Despite differences at the DNA and virus production levels, SRLV mRNA levels were not affected by SeV infection. Estimated SRLV proviral load in vivo is around one copy per cellular genome, ensuring low protein production and immune system evasion [49]. High LTR transcription promoter activity is likely to ensure high SRLV transcription rates even at low proviral load conditions [50]. This may explain the lack of statistical significance of SRLV viral RNA levels between uninfected and SeV-stimulated cells.

SeV infection induces an anti-SRLV restriction in cells already infected with SRLV (AM; Figure 3A) or in SRLV-free cells (BDM and OSF) that are experimentally infected with SRLV (Figure 3B), thereby showing therapeutic and prophylactic potentialities, respectively. This is in agreement with previous observations linking proinflammatory responses with antiviral states not only in ovine macrophages [8,51,52], but also in humans [9,53]. An HIV-1 GFP-encoding vector production was also inhibited in ovine cells infected with SeV, indicating the induction of broadly active innate immune responses. In addition, HIV-GFP vector showed a decreased infection of OSF previously infected with SeV-GFP (Figure 4). Similarly, HIV-GFP infectivity was also decreased when produced in SeV-GFP infected human 293-T cells, suggesting that, in addition to antigen specific responses, innate responses triggered by SeV in ovine and human cells can contribute to HIV-1 inhibition.

Remarkably, SeV-GFP infection also triggers the secretion of antiviral factors in ovine AM with paracrine effects. Supernatant transfer from SeV-infected AM to fresh OSF reduced SRLV virus production in these cells (Figure 6). The presence of type-I IFN in these supernatants could explain the induction of restriction factors in OSF as well as the activation of the antiviral programs leading to the anti-SRLV patterns observed. For example, BST2 is an ISG that was increased in OSF treated with supernatant from SeV-infected cells. In accordance, gene expression of Newcastle disease virus (NDV), another related paramyxovirus, in baby hamster kidney cells (BHK-21) also induced type-I IFN with paracrine effects on human PBMCs [54] Likewise, type-I IFN secreted from dendritic cells (DCs) infected with herpes simplex virus type-1 (HSV-1) mediates bystander activation of neighbor uninfected DCs [55].

The induction of antiviral programing in SeV-infected cells that leads to SRLV and HIV-GFP restriction is indicative of a non-specific antiviral induction state, which could be convenient when aiming to induce a response against different SRLV strains. These induction properties could be enhanced by the expression of SRLV recombinant genes using SeV as a vector. SeV vectors may afford the introduction of genetic regions about 4 Kb long, which is the length of some lentivirus structural proteins. The generation of recombinant SeV with the ability to overexpress SRLV proteins could be good vaccine candidates.

Infection of sheep with either SeV or transmission incompetent ΔF/SeV has already been proven to be efficient using vibrating mesh-based single-pass nebulizer or polyethylene catheters. This method could be used for infection and transgene expression in the lungs of the animals. In accordance with our results in vitro, no infectious SeV was detected in vivo. Furthermore, the use of this system guarantees a high SeV recombinant protein expression [19] based on our observations. Innate immunity stimulation and proper antigen presentation are well documented in various animal models using SeV vectors [56]. SeV-transduced dendritic cells induce persistent natural killer (NK) and CD4 anti-tumoral activity, which prevented metastasis [57]. These features justify further investigation in the use of SeV recombinant vaccine vectors for immunization against SRLV or other animal lentiviruses.

## 5. Conclusions

Development of vaccines against SRLV has been classically centered on the stimulation of adaptive immune responses with results ranging from disease enhancement to partial protection against SRLV homologous strains, therefore, no vaccine is currently available. Our data suggest that innate immunity can be induced in ovine cells through SeV-GFP infection. Ovine cells were efficiently infected by a SeV-GFP vector which trained immune response to counteract SRLV infection in experimentally and naturally infected cells. Antiviral state is characterized by the expression of intrinsic restriction factors that target homologous (SRLV) and heterologous (HIV-1) lentiviruses. Finally, this antiviral activity can be likely transferred, because of type-I IFN production, to new cells in a paracrine manner.

## Supplementary Materials

The following are available online at https://www.mdpi.com/2076-393X/8/2/206/s1, Figure S1:Sendai virus vector expressing GFP infection of ovine skin fibroblasts (OSF) at different multiplicities of infection (MOI), Figure S2: Sendai virus vector expressing GFP (SeV-GFP) is transmission-deficient in ovine cells, Figure S3: Small Ruminant Lentivirus (SRLV) kinetics after infection of ovine skin fibroblasts (OSFs) previously infected with Sendai virus vector (SeV), Figure S4: Small Ruminant Lentivirus (SRLV) restriction in permissive cell lines, T-immortalized goat embryo fibroblasts (TIGEF) and goat synovial membrane cells (GSM-T).
