# Supplementary material for: Sendai Virus, a Strong Inducer of Anti-Lentiviral State in Ovine Cells
Source: Vaccines (Basel). 2020 Apr 29;8(2):206. doi: 10.3390/vaccines8020206 (PMC7349755; doi:10.3390/vaccines8020206)
Supplement: Supplementary file 1 [file vaccines-08-00206-s001.pdf]

**Supplementary Materials:**

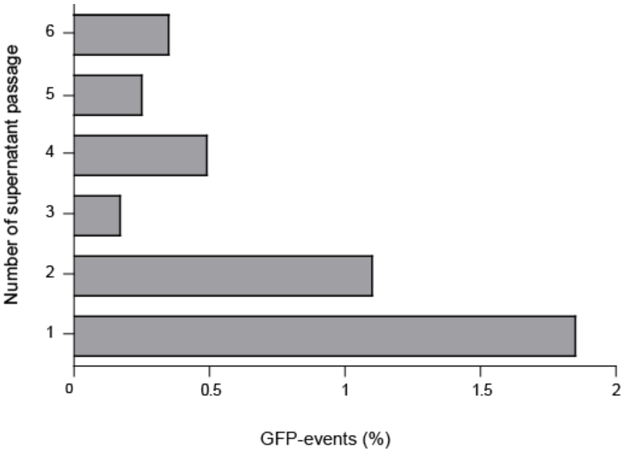


**Figure S1.** Sendai virus vector expressing GFP infection of ovine skin fibroblasts (OSF) at different multiplicities of infection (MOI). Data represent GFP-positive events measured by flow cytometry.

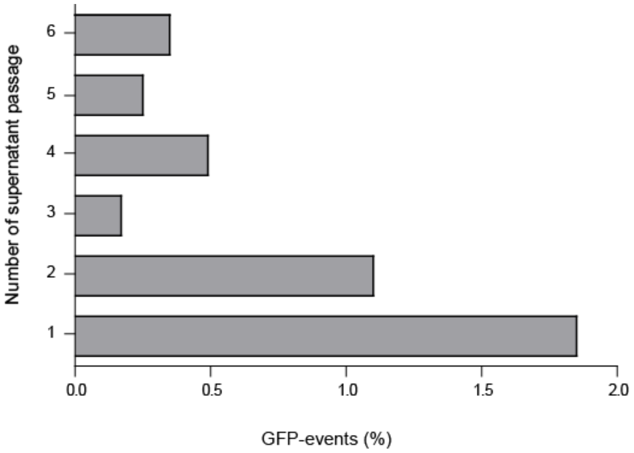


**Figure S2.** Sendai virus vector expressing GFP (SeV-GFP) is transmission-deficient in ovine cells. Supernatants from ovine skin fibroblasts (OSF) infected with SeV-GFP were serially transferred to uninfected OSF and GFP-positive events counted in a flow cytometer.

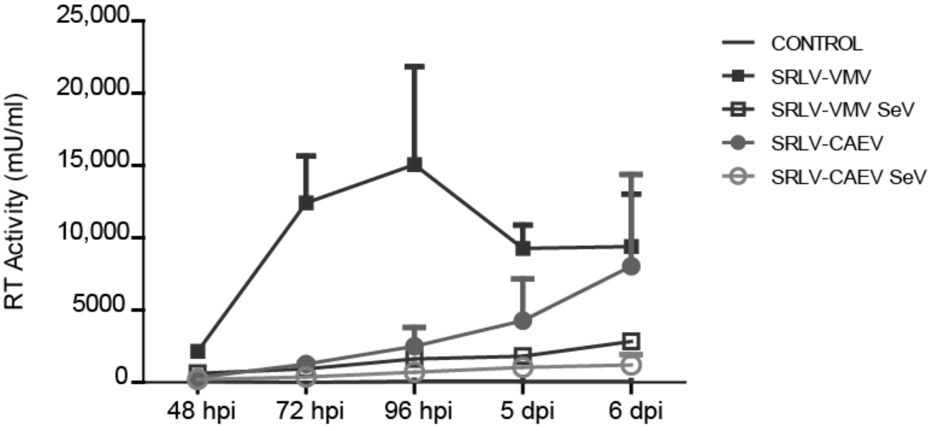

**Figure S3.** Small ruminant lentivirus (SRLV) kinetics after infection of ovine skin fibroblasts (OSFs) previously infected with Sendai virus vector (SeV). RT activity was measured in the supernatants at different hours (hpi) or days (dpi) post-infection with SRLV from the genotypes A (VMV) or B (CAEV).

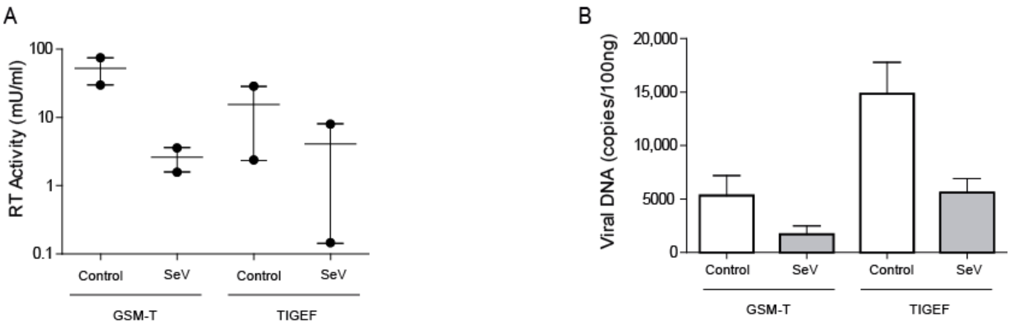


**Figure S4.** Small ruminant lentivirus (SRLV) restriction in permissive cell lines, T-immortalized goat embryo fibroblasts (TIGEF) and goat synovial membrane cells (GSM-T): (**A**) Retrotranscriptase (RT) activity measured by SG-PERT in clarified supernatants from cells infected with SRLV (control) and infected with Sendai virus vector (SeV). The plots display median values ±interquartile range. (**B**) Viral DNA measured 16 h post-infection with SRLV at 0.5 MOI in control GSM-T and TIGEF cells (white bars) and infected with SeV (grey bars). Values are the median (±interquartile range). Experiments were performed twice, thus statistical analysis was not performed.
